# Supplementary material for: Patterns of Intron Gain and Loss in Fungi
Source: PLoS Biol. 2004 Nov 30;2(12):e422. doi: 10.1371/journal.pbio.0020422 (PMC532390; doi:10.1371/journal.pbio.0020422)
Supplement: Table S1 — Also available at http://genes.mit.edu/NielsenEtAl/. (4.3 MB ZIP). [file pbio.0020422.st001.zip › NielsenEtAl/html/81.html]

AN4718.1.NCU00667.1.MG03065.1.FG08573.1


```
 CLUSTAL W (1.82) Multiple Sequence Alignments - Introns Inserted


Sequence 1: NCU00667.1	167 aa
Sequence 2: FG08573.1	160 aa
Sequence 3: MG03065.1	168 aa
Sequence 4: AN4718.1	237 aa
Alignment Length: 237 aa
Number Identitical Residues: 107 aa
Alignment Score (without introns) 4525


MG03065.1 	---------------------------------------~--------------------
NCU00667.1	---------------------------------------~--------------------
FG08573.1 	---------------------------------------~--------------------
AN4718.1  	MDWPVERSSLDNEHQTKTSSKGNEVAVEGITGSRGWECL1SLIETRAILQLVSSISNLPS
          	 . . . :: ... .:.:::.... : .. :.: . ..  :  .: :  .  :: :. .:

MG03065.1 	--MPGVVPDDSELAPKFAPFVGM0AGIAAA~MIFG-------C1AGAAFGTAKSGIGIAG
NCU00667.1	---MAEIMADSELAPKFAPFIGM0AGIAAA~MIFG-------S1AGAAYGTAKSGIGIAG
FG08573.1 	-------MAESELAPKFAPFIGM0AGIAAA~MIFG-------C1IGAAYGTAKSGIGIAG
AN4718.1  	LIKESITMTESELTPKFAPFLSF0AGIAAA0LAAESPRLTDTA~AGAAYGTAKAGIGIAG
          	    .    :***:******:.: ****** :   :.  :.:.  ***:****:******

MG03065.1 	VGTFRPDLIMK0CLIPVIMSGIIAVYALVVAVLIAQDLNAPT-AGTSYDLFR~GIMHLAC
NCU00667.1	VGTFRPDLIMK0CLIPVVMSGIIAVYALVVAVLIAQDLGPPG-SGQHYSLFN2GFMHLAC
FG08573.1 	VGTFRPDLIMK0CLIPVVMSGIIAVYSLVISVLIAEDLDPS----KNYSLFS2GFMHLGC
AN4718.1  	VGTFRPDLIMK0SLIPVVMSGIIAVYGLVIAVLIQGDVTAPTGSEPSLSLYA2GCMHLAS
          	*********** .****:********.**::***  *: .. .:    .*:  * ***..

MG03065.1 	GLSVGLTGLAAGYCIGIVGDK~GVRAYMEQSRIFVGMVLILIFGEVLGLYG2LIVALILN
NCU00667.1	GLSVGLTGLAAGYCIGIVGDK~GVRSFMLQSRIFVGMVLILIFGEVLGLYG2LIVALILN
FG08573.1 	GIAVGMTGLAAGYCIGIVGDT~GVRAYMEQSRIFVGMVLILIFGEVLGLYG2LIVALILN
AN4718.1  	GLSVGLAGIAAGYTIGIVGDA0GVRAYMQQSRVYVGMILILIFGEVLGLYG2LIVGLILN
          	*::**::*:**** ******  ***::* ***::***:************* ***.****

MG03065.1 	TRSQE
NCU00667.1	TKSKG
FG08573.1 	TKSKG
AN4718.1  	SKSSN
          	::*.
```
